# Vaccination: A Consideration of Some Points as to the Indentity of Variola and Vaccinia

**Published:** 1881-11

**Authors:** Henry M. Lyman

**Affiliations:** Chicago, Ill.


### ARTICLE VI.

### VACCINATION: A CONSIDERATION OF SOME POINTS AS TO THE IDENTITY OF VARIOLA AND VACCINIA. By HENRY M. LYMAN, A.M., M.D., Chicago, Ill.

I have read with much interest the article under the above title, by Dr. Thomas F. Wood, in the October number of THE CHICAGO MEDICAL JOURNAL AND EXAMINER. It presents in an excellent manner the objections to the hypothesis of the identity of variola and vaccinia, reinforcing the same with a number of original observations and experiments by the author. His conclusions are essentially those which have been formulated by Professor Chauveau, and have been frequently reiterated by Dr. Henry A. Martin.

It is, in the first place, very evident that the author of the paper under consideration is somewhat embarrassed by the multitude of his unclassified facts. The meaning to be attached to the words *identity* is also a source of confusion. It should be kept in mind that in a discussion regarding scientific classifications in natural history, the words *identity* and *identical* are used in at least two ways. It is right to speak of objects as being *generically* identical, though they are *specifically* distinct and different. In the same way objects or creatures which are specifically identical are nevertheless differentiated into well defined varieties. Domestic sheep, for instance, are all identical in their species, but there are numerous varieties—as Merinos, Southdowns, Cotswolds, etc.

Now variola presents itself as a disease which is capable of existing in numerous different species of animals, and its phenomena vary accordingly. I will not at present discuss the question whether such variations are sufficient to constitute different species, or whether the modifications should be only ranked as varieties of one species. For many reasons it will be convenient to use the term *genus* as applied to the original form of the disease. Small-pox, then, would be the generic form of the disease; horse-pox would constitute one species; cow-pox another; sheep-pox a third; and so on indefinitely. All these different forms of disease, having one common origin, would be generically the same, though specifically different from each other. Something of this sort is what is intended when we speak of the identity of vaccinia and variola.

Dr. Wood contends, however, that small-pox and cow-pox are not thus identical; and he adduces four objections upon which his opinion is based:

1. *Because cow-pox has never been converted by any means into small-pox.*

In reply to this I would say, that the efforts of experimenters have never yet been knowingly turned in this direction. The fact that a thing has never yet been done is no proof that it cannot be done. But, according to Dr. Wood himself (p. 354), the thing has been done with great success. If his experiment at the Wilmington Small-pox Hospital was not a case of conversion of cow-pox from the cow into small-pox in the child, it is impossible to say what it was.

2. *Because, in the light of the attempts made by many careful and truthful experimenters, to produce small-pox by the inoculation of the cow with small-pox virus, the number of failures on the part of such a very large majority of them, together with the numerous positive denials made of the possibility of success, it may be concluded that small-pox virus is never converted into vaccine by transmission through the cow.*

It is very true that many experimenters in this matter have failed again and again in their efforts to variolate the cow. But it is also true that when the correct method has been employed it has yielded a successful result. Witness the interesting experiments of Mr. Badcock, the English druggist, who, after many failures, finally taught himself the proper way to variolate the cow. He made use of the best variolous virus that his physician could furnish, and his experiments were successfully repeated until he had vaccinated 14,000 children with the resulting vaccine virus, and had supplied it to not less than 4,000 physicians. Such results do not look like failure to procure vaccine virus through variolation of the cow.

3. *Cow-pox does not become epidemic, nor does it spread by*

*effluvia from the human subject. It is always confined to the point of insertion in the human subject, never producing a general eruption of vaccine vesicles.*

It is difficult to discover the relevancy of this proposition to the present discussion. Cow-pox never spreads by effluvia among cattle, even though it be the result of actual variolation with small-pox virus. This is one of the evidences of the modification of that virus by cultivation in the body of the cow. The statement that cow-pox never produces a general eruption of vaccine vesicles is a little too sweeping. Active vaccine virus does occasionally produce a somewhat general eruption, just enough to show its kinship to variola.

4. *Structurally considered, the vesicles of vaccination and the pustules of inoculation are very dissimilar.*

This fact cannot be urged against the identity of cow-pox. The structure of the vesicle depends upon the kind of animal on which it is developed, and upon the manner in which the virus is introduced into the body. By inoculation with variolous virus a pustule is developed on the human body which is almost identical in appearance and in its course with the results of a similar inoculation on a horse or a cow. In fact the vesicle of ordinary cow-pox is really an inoculation vesicle, for the cow seldom contracts the disease in any other way than by direct insertion of the virus, either accidentally or by design. The energy of the virus which is thus introduced is so weakened by the local processes of vesiculation that it does not produce any extensive eruption like that which often follows inoculation with small-pox virus in the human subject. This fact shows that the complete evolution of small-pox is subject to modification in accordance with the nature of the animal in which the process is carried out.

The great source of difficulty and obscurity with authors like Chauveau, Martin, and our friend Dr. Wood, lies in the fact that they do not perceive that variolous virus, like all other forms of virus, is a very mutable substance. It is continually liable to dilution and reduction of energy. It may again be vivified and made more energetic, once more to experience attenuation; and so on indefinitely. Trousseau's experiments, as related in his interesting chapter on cow-pox, are remarkably instructive. Inoculating children with small-pox virus, and transmitting the virus from one to another in successive "cultivations," he found a continual tendency to milder manifestations of the disease in each successive crop. But occasionally there would be a reversion to the original type, and a child which had been inoculated with a mild form of the virus would be attacked with a malignant form of small-pox. The cause of such reversions was not discovered by Trousseau, and I will not now delay for its explanation. I simply wish at present to call attention to the progressive mitigation of the disease which is the ordinary result of its artificial propagation. This mitigation is still more apparent when the disease is communicated from man to the lower animals. According to the experiments of Thielé, such mitigation of the virus may be obtained without cultivation in the body of any animal whatever, by simply diluting the virus with milk after keeping it for ten or twelve days between two glasses. Dr. Wood quotes this observation (p. 350) only to discredit it. We might very easily do so, were it not that the recent experiments of Pasteur with anthrax virus render the statements of Thielé perfectly reasonable and probable. This subject, however, will soon be experimentally investigated by Burdon-Sanderson, for whose results we can wait.

Recognizing the fact that small-pox virus is capable of attenuation by successive cultivations, we can easily comprehend the manner in which such apparently irreconcilable experiments as those of Gassner, Thielé, Ceely and Badcock can be harmonized with the equally trustworthy observations of Chauveau, Wood, and others. When small-pox virus is first inoculated upon the cow, the product is a virus but one degree removed from its original intensity. If such virus be used for vaccination of the human infant, the result will either be small-pox, as Chauveau and Wood saw, or it will produce a very severe form of vaccine-disease, as was the experience of Ceely. But if this virus be still further attenuated by repeated transmissions from one cow to another, or from horse to cow, as was so common during the centuries before Jenner, the virus will be so far diluted that it will never give origin to small-pox when transplanted to the human subject. In ike manner, Ceely found that the virus which he produced by variolation of the cow, though exceedingly energetic at first, even lost its dangerous energy by successive transmission from one child to another, so that after the fourth or fifth remove from the cow it was perfectly manageable, and as mild as ordinary Jennerian virus.

As for the origin of cow-pox in the cow, it is now recognized that, whatever its mediate source may be, its primitive source is small-pox. Derived from horse-pox, it only occurs when the milker of the cow is also the attendant upon a greasy horse. Sometimes, but rarely in these days, it may be derived from inhalation of air charged with variolous human excreta. When neither of these sources of contagion are active it may be traced to contact with the products of human vaccination among the milkers. Thus it is evident that, besides artificial variolation, there are at least three ways by which cow-pox may be originated in the cow. The resulting disease is characterized in every case by the same phenomena, showing that the species is the same notwithstanding the diversity of the mediate sources through which it was evolved. Cow-pox is cow-pox, no matter what may have been its immediate antecedent. But that it constitutes a species with varieties of individual manifestation is evident from the diversity of its intensity when transplanted into the human subject—a diversity which, as we have seen, is largely dependent upon the antecedent history of each specimen of the virus. Moreover, since the tendency of successive transmissions of virus though the cow is in the direction of mitigation of the successive crops, it follows that in time the product of each transmission must deteriorate just as vaccine-virus has deteriorated in the human subject. It will then be necessary to revert once more to a point nearer the original variolous source, in order to recruit the energies of our vaccine stock. This we can always accomplish in future by variolation of the cow, if Beaugency heifers cannot be discovered when needed. Guided in the preparation of such virus by the experience of the past, it will be an easy matter to avoid the difficulties and the dangers which lead men like Dr. Wood to entertain no little esteem for such a means of renewing the energy of vaccine virus.

There are many other interesting and practical thoughts suggested by consideration of this subject, but I have not time for their presentation. To those who are interested in this important matter, I can recommend perusal of Trousseau's fascinating chapter on Cow-pox, the article on Vaccination, in the seventh volume of the Real-Encyclopädie der Gesammten Heilkunde, the various reports on anthrax vaccination by Pasteur, Greenfield, Toussaint, and others; and, lastly, an essay on Disease Germs, by Dr. W. B. Carpenter, in the October number of *The Nineteenth Century*.

